# Alveolar–arterial oxygen gradient nonlinearly impacts the 28‐day mortality of patients with sepsis: Secondary data mining based on the MIMIC‐IV database

**DOI:** 10.1111/crj.13614

**Published:** 2023-04-19

**Authors:** Ying Wang, Yan He, Lu Chen, Ying Liu, Jia Yuan, Hongying Bi, Qimin Chen, Xianjun Chen, Feng Shen

**Affiliations:** ^1^ Department of Intensive Care Unit The Affiliated Hospital of Guizhou Medical University Guiyang China; ^2^ Clinical Trial Centre The Affiliated Hospital of Guizhou Medical University Guiyang China

**Keywords:** D(A‐a)O_2_, MIMIC‐IV database, mortality, sepsis

## Abstract

**Objective:**

Lung is often implicated in sepsis, resulting in acute respiratory distress syndrome (ARDS). The alveolar–arterial oxygen gradient [D(A‐a)O_2_] reflects lung diffusing capacity, which is usually compromised in ARDS. But whether D(A‐a)O_2_ impacts the prognosis of patients with sepsis remains to be explored. Our study aims to investigate the association between D(A‐a)O_2_ and 28‐day mortality in patients with sepsis using a large sample, multicenter Medical Information Mart for Intensive Care (MIMIC)‐IV database.

**Methods:**

We extracted a data of 35 010 patients with sepsis from the retrospective cohort MIMIC‐IV database, by which the independent effects of D(A‐a)O_2_ on 28‐day death risk was investigated, with D(A‐a)O_2_ as being the exposure variable and 28‐day fatality being the outcome variable. Binary logistic regression and a two‐piecewise linear model were employed to explore the relationship between D(A‐a)O_2_ and the 28‐day death risk after confounding factors were optimized including demographic indicators, Charlson comorbidity index (CCI), Sequential Organ Failure Assessment (SOFA) score, drug administration, and vital signs.

**Results:**

A total of 18 933 patients were finally included in our analysis. The patients' average age was 66.67 ± 16.01 years, and the mortality at 28 days was 19.23% (3640/18933). Multivariate analysis demonstrated that each 10‐mmHg rise of D(A‐a)O_2_ was linked with a 3% increase in the probability of death at 28 days either in the unadjusted model or in adjustment for demographic variables (Odds ratio [OR]: 1.03, 95% CI: 1.02 to 1.03). But, each 10 mmHg increase in D(A‐a)O_2_ was associated with a 3% increase of death (OR: 1.03, 95% CI: 1.023 to 1.033) in the case of adjustment for all covariants. Through smoothed curve fitting and generalized summation models, we found that non‐linear relationship existed between D(A‐a)O_2_ and the death at 28‐day, which demonstrated that D(A‐a)O_2_ had no any impacts on the prognosis of patients with sepsis when D(A‐a)O_2_ was less than or equal to 300 mmHg, but once D(A‐a)O_2_ exceeded 300 mmHg, however, every 10 mmHg elevation of D(A‐a)O_2_ is accompanied by a 5% increase of the 28‐day death (OR: 1.05; 95% CI:1.04 to 1.05, *p* < 0.0001).

**Conclusion:**

Our findings suggests that D(A‐a)O_2_ is a valuable indicator for the management of sepsis patient, and it is recommended that D(A‐a)O_2_ be maintained less than 300 mmHg as far as possible during sepsis process.

## Graphical abstract

D(A‐a)O_2_ is a valuable indicator for the management of sepsis patient. When D(A‐a)O_2_ was less than or equal to 300 mmHg, it has no any association with the prognosis of patient, but the mortality rate increases once D(A‐a)O_2_ became greater than 300 mmHg. The safe range of D(A‐a)O_2_ for patients with sepsis in the hospital and ICU is less than 300 mmHg.

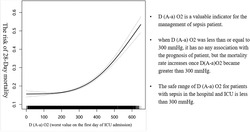

## 1 INTRODUCTION

Sepsis is one of the most severe disease processes in clinical settings. The disease has been paid more and more attention to by clinicians since 1991, and Surviving Sepsis Campaign (SSC) guideline has been updated several times since its first version in 2001,^1^, ^2^ among which the most recent definition of sepsis is life‐threatening organ dysfunction because of a dysregulated host response to infection.^2^ Sepsis is estimated to affect more than 19 million people each year, killing approximately 5 million worldwide.^3^ Despite substantial advances in understanding the host response to microbial pathogens, sepsis mortality has unfortunately not decreased so far. One of the bottlenecks is the lack of valid model to effectively predict the prognosis of sepsis and thus provide a basis for prognostic enrichment, leaving clinical trials of new drugs with insufficient power.^4^

The alveolar–arterial oxygen gradient (D[A‐a]O_2_) is a specific indicator reflecting the diffusion capacity of lung function, with a normal physiologic range of 7–14 mmHg^5^ in healthy adults (fraction of inspired oxygen [FiO_2_] being 21%), which is also associated with age. The capacity has been proven to be impaired in some conditions such as severe pulmonary, especially in acute respiratory distress syndrome (ARDS). Clinical studies reported that D(A‐a)O_2_ could identify COVID‐19 patients at risk of developing severe pneumonia early^6^ and also has the ability to predict mortality rate of interstitial pneumonia in patient with dermatomyositis treated by cyclosporine A/glucocorticosteroid combination therapy.^7^ Meanwhile, D(A‐a)O_2_ could predict the short‐term prognosis in patients with submassive pulmonary embolism and is useful in risk stratification of these patients.^8^ During sepsis, the lungs are one of the most involved organs, making sepsis‐related ARDS to be a common complication.^9^, ^10^ Occurrence of ARDS in turn aggravates the severity of sepsis because of its many pathologic characteristics, of which diffusion capacity is severely decreased (indicated as D[A‐a]O_2_ value increase), resulting in oxygenation dysfunction, a pivotal mechanism for sepsis genesis.^11^ This mechanism plays a pivotal role in sepsis pathogenesis, suggesting that D(A‐a)O_2_ may be linked to sepsis prognosis.

The goal of our study is to investigate the relationship between D(A‐a)O_2_ and the risk of 28‐day mortality in sepsis patient basing on the Medical Information Mart for Intensive Care (MIMIC)‐IV large sample sepsis database from the United States. A larger sample size would provide more consistent and trustworthy results, enabling us to gain a better understanding of the relationship between these two variables in sepsis.

## 2 METHODS

### 2.1 Source of information

The data was sourced from the MIMIC‐IV database, which was designed to be applied in retrospective cohort research, including clinical data of patients treated at Beth Israel Deaconess Medical Center (BIDMC) from 2008 to 2019. The database is free to be downloaded once one completes an approved course on the official website. Lu Chen, one of our authors, has finished the certified course and acquired access to the database, and she hence is in charge of data extraction (record ID: 50668217). Our analysis conforms to the RECORD (REporting of Studies Conducted Using Observational Routinely‐Collected Health Data) statement.

### 2.2 Queue information

The MIMIC‐IV database includes 377 207 adult patient records in total, from which we extracted data of 35 010 ones with sepsis diagnosis. The extraction was based on the ICD‐9 and ICD‐10 codes recorded in the database (ICD‐9 codes 99591‐99592 or ICD‐10 codes R652, R6520, and R6521), spanning from 2008 to 2019. All of these patients whose data were extracted received treatment at BIDMC. We examined the interaction between 28‐day death and D(A‐a)O_2_ value, with 28‐day death as the outcome variable (dichotomous *Y* = 1, death; *Y* = 0, survival) and the D(A‐a)O_2_ value as the exposure variable (recorded as a continuous variable). The worst D(A‐a)O_2_ values from the first day of ICU admission were extracted for analysis. Patients with missing exposure variable information were not included in this study.

Based on our clinical experience and the literature,^12^, ^13^ we chose these covariates including gender (male or female), age (years), ethnicity, Charlson comorbidity index (CCI); Sequential Organ Failure Assessment (SOFA) score, PO_2_/FiO_2_ value, heart rate and body temperature at admission; use of mechanical ventilation, glucocorticoids (dexamethasone, methylprednisolone, hydrocortisone), vasoactive drugs (dopamine, dobutamine and norepinephrine), immunoglobulins, and antibiotic administration (carbapenems, cephalosporins, penicillins, and vancomycins).

### 2.3 Statement on ethics and informed consent

The MIMIC‐IV database was authorized by the institutional review boards of BIDMC (2001‐P‐001699/14) in Boston, Massachusetts, and the Massachusetts Institute of Technology (0403000206). It is now available on the internet. Patient's informed permission was revoked as the data is publicly available, and the patient's identifiable information is uncertain.

### 2.4 Description of missing data

Multiple interpolation was not utilized to fill in the blanks as the missing rate of the variables used in this study was less than 5% (0–4.1%).

### 2.5 Statistical analysis

Continuous variables were expressed as the mean + standard deviation (Gaussian distribution) or as the median (minimum, maximum) (skewed distribution). Categorical variables were presented as rate (percentage). Considering that it was a cohort study, we categorized exposure variables into four groups (quartiles, Q1 to Q4) and examined the distribution of patient baseline information across the different subgroups. To evaluate any statistical differences between the means and proportions of the groups, we performed one‐way ANOVA (Gaussian distribution), Kruskal–Wallis H (skewed distribution), and chi‐square tests (categorical variables). Meanwhile, we also employed univariable and multivariable binary logistic regression models to investigate the relationship between D(A‐a)O_2_ and 28‐day mortality under four different models, that is, Model 1 (no factors were adjusted), Model 2 (adjusted for demographic characteristics only) and Model 3 (adjusted for all covariates presented in Table 1). Finally, we have done a series of sensitivity analyses: (1) We converted D(A‐a)O_2_ from a continuous to a categorical variable (quartile, Q1 to Q4) and calculated *P* for the trend of relationship. The purpose of sensitivity analysis was to determine whether the results of categorical variable were as robust as those of the continuous variable. (2) We employed several different adjustment strategies to evaluate the robustness of our results.

**TABLE 1:** Characteristics of patients at baseline.

D(A‐a)O_2_ (mmHg)	Q1(0.00–148.1)	Q2(148.1–230.4)	Q3(230.4–331.4)	Q4(331.5–649.7)	*p*‐value
	(N = 4733)	(N = 4731)	(N = 4734)	(N = 4735)	
Age at admission, mean ± sd, year	64.74 ± 16.81	66.07 ± 15.61	66.30 ± 14.50	65.26 ± 15.34	<0.001
Male sex, n (%)	2031 (42.91%)	1952 (41.26%)	1722 (36.38%)	1776 (37.51%)	<0.001
White, n (%)	2981 (62.98%)	3129 (66.14%)	3314 (70.00%)	3131 (66.12%)	<0.001
Charlson Comorbidity Index, mean ± sd, points	5.67 ± 3.02	5.79 ± 2.88	5.65 ± 2.69	5.92 ± 2.86	<0.001
SOFA score, mean ± sd, points	6.47 ± 3.49	7.29 ± 3.77	7.82 ± 3.85	9.37 ± 4.21	<0.001
Heart rate, mean ± sd, bts/min	103.72 ± 23.77	104.12 ± 23.88	103.16 ± 23.26	108.31 ± 25.71	<0.001
Body temperature, mean ± sd, °C	36.75 ± 1.38	36.73 ± 1.40	36.64 ± 1.43	36.72 ± 1.49	0.002
PO_2_/FiO_2_	372.52 ± 102.97	275.98 ± 91.20	235.38 ± 73.61	152.82 ± 61.09	<0.001
The use of dopamine, n (%)					<0.001
No	4465 (94.34%)	4431 (93.66%)	4406 (93.07%)	4250 (89.76%)	
Yes	268 (5.66%)	300 (6.34%)	328 (6.93%)	485 (10.24%)	
The use of dobutamine, n (%)					<0.001
No	4579 (96.75%)	4524 (95.62%)	4537 (95.84%)	4467 (94.34%)	
Yes	154 (3.25%)	207 (4.38%)	197 (4.16%)	268 (5.66%)	
The use of norepinephrine					<0.001
No	3085 (65.18%)	3120 (65.95%)	3074 (64.93%)	2493 (52.65%)	
Yes	1648 (34.82%)	1611 (34.05%)	1660 (35.07%)	2242 (47.35%)	
The use of dexamethasone, n (%)					<0.001
No	4190 (88.53%)	4278 (90.42%)	4362 (92.14%)	4334 (91.53%)	
Yes	543 (11.47%)	453 (9.58%)	372 (7.86%)	401 (8.47%)	
The use of methylprednisolone, n (%)					<0.001
No	3794 (80.16%)	3908 (82.60%)	4056 (85.68%)	3886 (82.07%)	
Yes	939 (19.84%)	823 (17.40%)	678 (14.32%)	849 (17.93%)	
The use of hydrocortisone, n (%)					0.027
No	4651 (98.27%)	4659 (98.48%)	4671 (98.67%)	4636 (97.91%)	
Yes	82 (1.73%)	72 (1.52%)	63 (1.33%)	99 (2.09%)	
The use of immunoglobulin, n (%)					<0.001
No	4621 (97.63%)	4644 (98.16%)	4654 (98.31%)	4603 (97.21%)	
Yes	112 (2.37%)	87 (1.84%)	80 (1.69%)	132 (2.79%)	
The use of carbapenems, n (%)					<0.001
No	3819 (80.69%)	3826 (80.87%)	3940 (83.23%)	3677 (77.66%)	
Yes	914 (19.31%)	905 (19.13%)	794 (16.77%)	1058 (22.34%)	
The use of cephalosporins, n (%)					0.111
No	4365 (92.22%)	4385 (92.69%)	4348 (91.85%)	4408 (93.09%)	
Yes	368 (7.78%)	346 (7.31%)	386 (8.15%)	327 (6.91%)	
The use of penicillins, n (%)					<0.001
No	2345 (49.55%)	2382 (50.35%)	2802 (59.19%)	2338 (49.38%)	
Yes	2388 (50.45%)	2349 (49.65%)	1932 (40.81%)	2397 (50.62%)	
The use of vancomycin, n (%)					<0.001
No	1024 (21.64%)	949 (20.06%)	966 (20.41%)	626 (13.22%)	
Yes	3709 (78.36%)	3782 (79.94%)	3768 (79.59%)	4109 (86.78%)	
The use of mechanical ventilation					<0.001
No	1100 (23.24%)	1134 (23.97%)	1179 (24.90%)	1456 (30.75%)	
Yes	3633 (76.76%)	3597 (76.03%)	3555(75.10%)	3279 (69.25%)	
28‐day‐mortality, n (%)					<0.001
survival	3992 (84.34%)	3944 (83.37%)	3926 (82.93%)	3431 (72.46%)	
Non‐survival	741 (15.66%)	787 (16.63%)	808 (17.07%)	1304 (27.54%)	

*Note*: Continuous data (mean ± SD), Categorical data (%).

Abbreviation: SOFA score: Sequential Organ Failure Assessment score.

As D(A‐a)O_2_ is a continuous variable, it could not be excluded that a nonlinear association existed between D(A‐a)O_2_ and 28‐day mortality. Therefore, we performed the generalized additive models (GAM) and smoothed curve fitting to investigate the connection between D(A‐a)O_2_ and sepsis 28‐day mortality. If the nonlinear relationship dose exist, we will explore the inflection point value by using a recursive technique then utilize the two‐piecewise linear model to obtain the OR value and 95% confidence interval on both sides of the inflection point.

All analyses were carried out with the statistical programs R (http://www.r-project.org, The R Foundation) and EmpowerStats (http://www.empowerstats.com, X&Y Solutions, Inc, Boston, MA). *P* value of less than 0.05 (two‐sided) is considered to be statistically significant.

## 3 RESULTS

### 3.1 Description of the patient screening process

**FIGURE 1:**
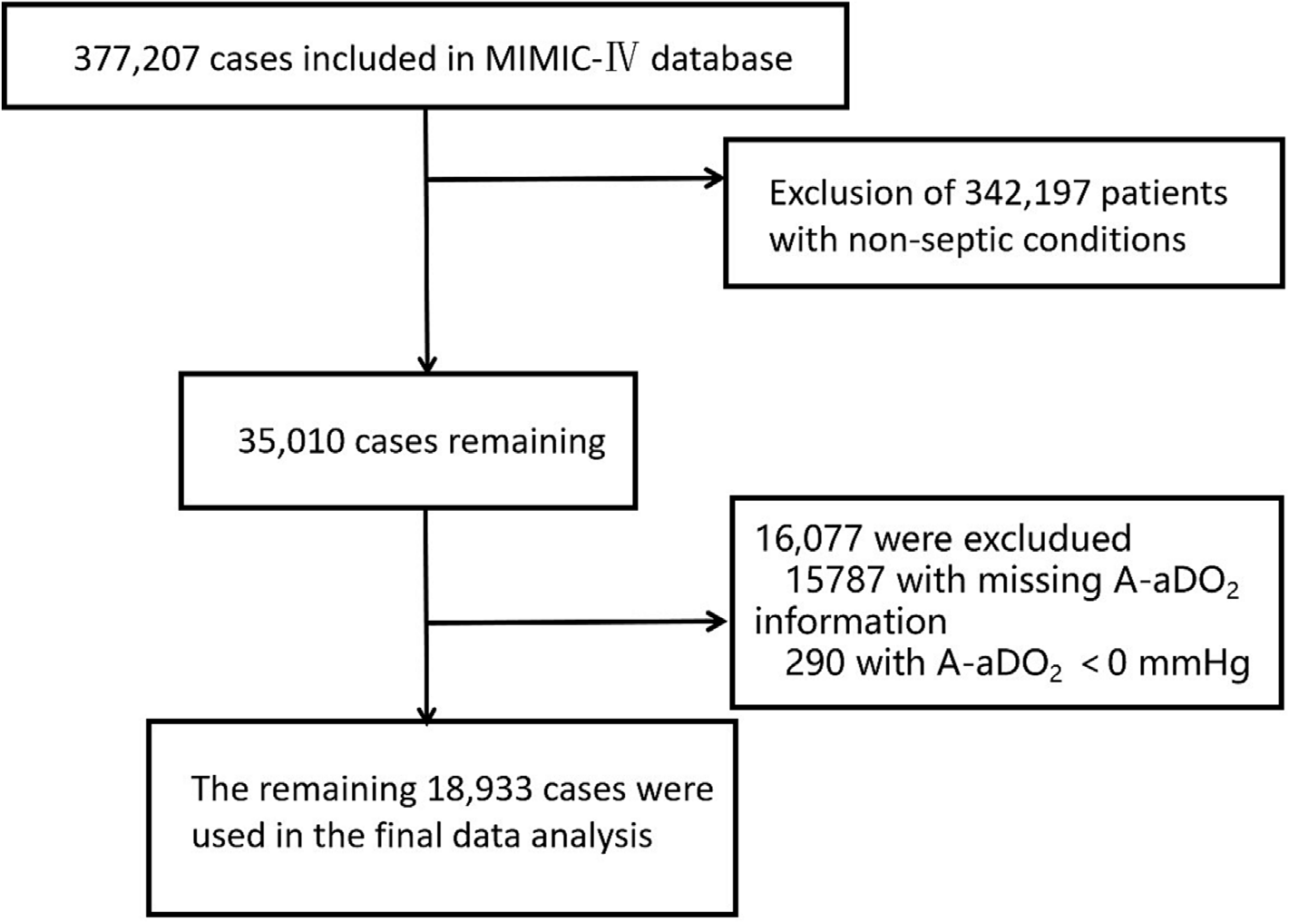
Flow chart of patient selection.

The MIMIC‐IV database contains data of 377 207 patients, among which the data of 342 197 patients were excluded because of non‐sepsis conditions. Among these, leaving the data of 35 010 patients, the data of 16 077 patients were also excluded as 15 787 of them are without D(A‐a)O_2_ information and another 290 are with D(A‐a)O_2_ value of less than 0. Ultimately, the data of 18 933 patients were included for the final analysis. (Figure 1).

### 3.2 Patient baseline characteristics

In order to observe the association between D(A‐a)O_2_ as categorical variable and 28‐day mortality, we categorized D(A‐a)O_2_ into four groups (Q1 to Q4) (Table 1). We observed the tendencies in the distribution of each variable among the different subgroups. The patients' average age was 66.67 ± 16.01 years. The fatality rate at 28 days was 19.23% (3640/18933).

Patients in Q4 group had higher Charlson co‐morbidity index, SOFA scores, heart rates, administration of intravenous gammaglobulin, hydrocortisone, carbapenem antibiotics, vancomycin, penicillin antibiotics, dobutamine, norepinephrine, and a 28‐day mortality when compared with the Q1, Q2, and Q3 groups, but has a lower value of PO2/FiO2 and received lower cephalosporin proportions than the Q1, Q2, and Q3 group. Patients in Q2 and Q3 group were older and had a lower rate of methylprednisolone use compared with the Q4 group. When compared to the Q4 group, patients in Q1 and Q2 group had higher rates of dexamethasone use.

### 3.3 Univariate and multivariate analyses for the association between D(A‐a)O_2_ and 28‐day mortality

In order to explore the relationship between D (A‐a)O_2_ and 28‐day death in patients with sepsis, we conducted univariate and multivariate analyses. Results indicated that each 10‐mmHg rise in D(A‐a)O_2_ was linked with a 3% increase in the probability of death at 28 days either in the unadjusted model or adjusted for variables in model 2 (Odds ratio (OR): 1.03, 95% CI: 1.02 to 1.03). In the adjustment of variables in Table 1, we found that each 10 mmHg increase of D(A‐a)O_2_ was accompanied with 3% of 28‐day mortality elevation (OR: 1.03, 95% CI: 1.023 to 1.033). In order to observe the trend testing, we conducted a sensitivity analysis, in which we converted D(A‐a)O_2_ into a categorical variable (quartile). The results demonstrated that when D(A‐a)O_2_ was treated as a categorical variable, the results were consistent with when D(A‐a)O_2_ was used as a continuous variable (Table 2). Furthermore, we investigated the influence of treatment strategies on outcomes, including the use of vasoactive drugs, glucocorticoids, and adaptation of antibiotic therapy. Our findings indicated that there was no significant change in effect values for D(A‐a)O_2_ with or without adjustment for the above variables (Supplemental table S1, S2 and S3).

**TABLE 2:** Results of univariate and multivariate analysis using non‐adjusted and adjusted Cox regression models.

Exposure	Model 1		Model 2		Model 3	
	OR, 95%CI	*p* value	OR, 95% CI	p value	OR, 95% CI	*p* value
D(A‐a)O_2_ ^a^	1.03 (1.02, 1.03)	<0.001	1.03 (1.02, 1.03)	<0.001	1.03 (1.023, 1.033)	<0.001
D(A‐a)O_2_ quartile^b^						
Q1	1.0		1.0		1.0	
Q2	1.08 (0.96, 1.20)	>0.05	1.07 (0.95, 1.19)	>0.05	0.80 (0.69, 0.93)	0.003
Q3	1.11 (0.99, 1.24)	>0.05	1.14 (1.02, 1.27)	<0.05	0.94 (0.80, 1.10)	0.438
Q4	2.05 (1.85, 2.27)	<0.001	2.12 (1.91, 2.35)	<0.001	1.69 (1.41, 2.02)	<0.001
P for trend	<0.001		<0.001		<0.001	

*Note*: Model 1: unadjusted model.Model 2: adjusted for gender, age at admission and ethnicity.Model 3: adjusted for variables that are presented in Table 1.

^a^ Continuous variable.

^b^ Categorical variable.

### 3.4 Results of the nonlinear association between D(A‐a)O_2_ and 28‐day mortality

**FIGURE 2:**
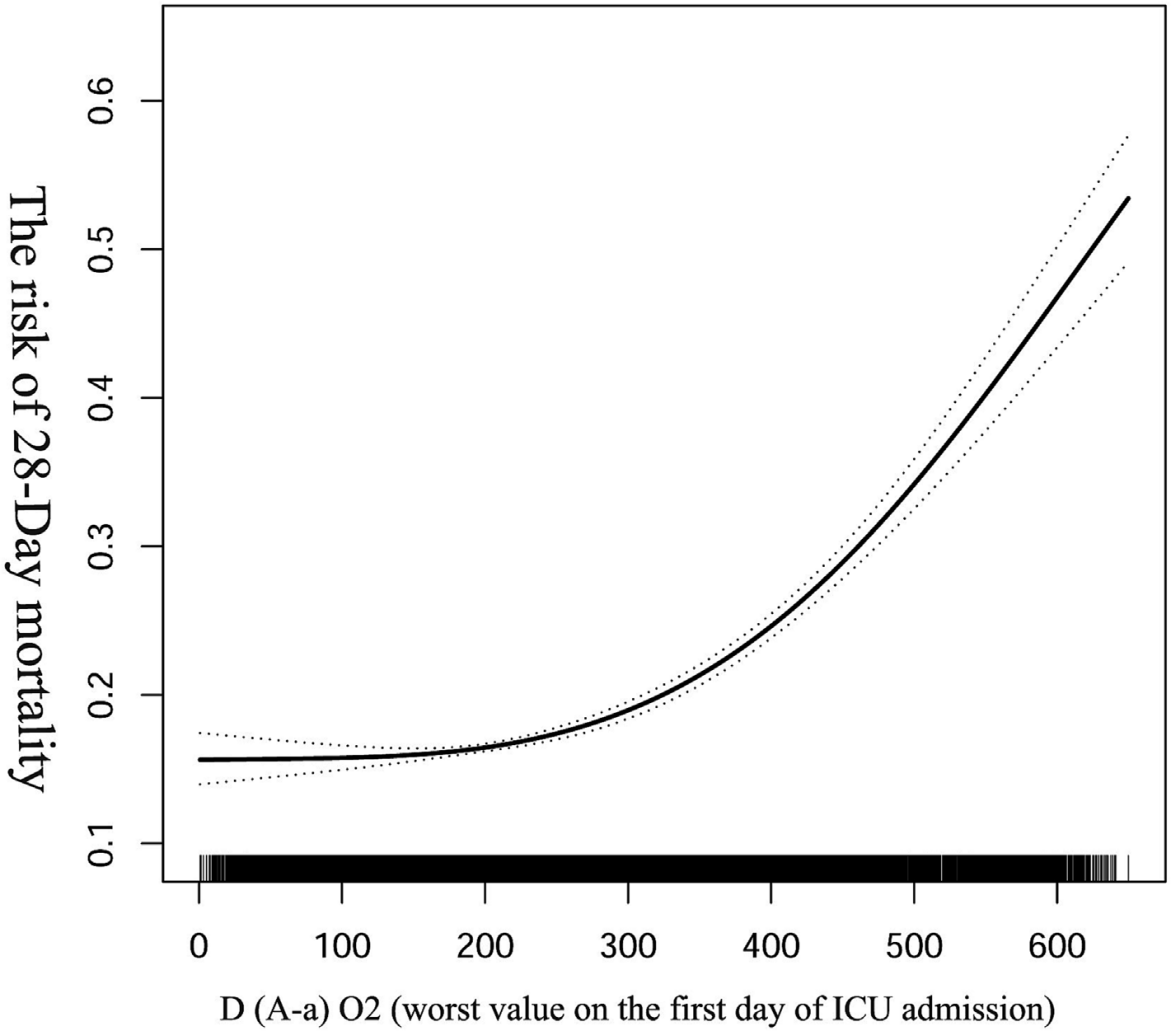
Results of the nonlinear association.

We used smoothed curve fitting and generalized summation models to investigate the relationship of D(A‐a)O_2_ and 28‐day death. After adjusting for all covariables shown in Table 1, analytic results showed a curvilinear relationship existing between D(A‐a)O_2_ and 28‐day mortality, which demonstrated that D(A‐a)O_2_ had no impacts on 28‐day mortality when D(A‐a)O_2_ was less than or equal to 300 mmHg; but, once D(A‐a)O_2_ value exceeded 300 mmHg, every 10 mmHg elevation of D(A‐a)O_2_ was associated with a 5% increase of mortality (OR: 1.05; 95% CI:1.04 to 1.05, *p* < 0.0001) (Figure 2, Table 3).

**TABLE 3:** Non‐linear relationships addressing.

28‐day mortality	HR(95%CI)	*p*‐value
Fitting model using standard logistic regression model	1.03 (1.023, 1.033)	<0.0001
Fitting model using two‐piecewise linear model		
Folding Points	300 mmHg	
≤300 mmHg	1.00 (1.00, 1.01)	0.0948
>300 mmHg	1.05 (1.04, 1.05)	<0.0001
Log‐likelihood ratio test	<0.001	

## 4 DISCUSSION

In this large retrospective cohort analysis, we discovered a curvilinear association between D(A‐a)O_2_ and 28‐day mortality in patients with sepsis, with an inflection point of 300 mmHg of D(A‐a)O_2_, which demonstrated that when D(A‐a)O_2_ was less than or equal to 300 mmHg, it has no association with the prognosis of the patient, but once D(A‐a)O_2_ became greater than 300 mmHg, each 10 mmHg increase is associated with a 5% increased risk of mortality (OR: 1.05; 95% CI: 1.04 to 1.05, *p* < 0.0001). As far as we know, few previous clinical studies have focused on the relationship between D(A‐a)O_2_ and the prognosis of sepsis patient.

D(A‐a)O_2_ refers to the difference between the alveolar and the arterial oxygen partial pressure, with the capability of hypoxia detection, which is mainly associated with pulmonary diffusion function, anatomical shunt, and so forth. In physiologic condition, D(A‐a)O_2_ will increase with the rise of concentration of inhaled oxygen, but rarely exceeds 56 mmHg.^5^ However, some respiratory disorders could make D(A‐a)O_2_ dramatically elevated, such as interstitial pneumonia, sever pulmonary fibrosis, and ARDS, in which diffusion dysfunction or ventilation/perfusion (V/Q) ratio mismatch occurred. D(A‐a)O_2_ is therefore often used as an indicator to measure the severity of these diseases. For patients with submassive pulmonary embolism, Ince O et al found that a D(A‐a)O_2_ of ≥42.38 mmHg had a good predictive value for a 90‐day mortality, with an area under curve of 0.83 and a sensitivity, specificity, and negative predictive value of 93.3%, 65.1%, and 98.6%, respectively.^8^ Pipitone et al reported that D(A‐a)O_2_ is superior to PaO_2_/FiO_2_ in identifying COVID‐19 patients at risk of developing severe pneumonia early, with an area under curve of 0.877 and a sensibility of 77.8%, a positive and negative predictive value of 75% and 94%, respectively, in the case of D(A‐a)O_2_ being ≥60 mmHg.^6^ However, our study found a significantly different “inflection point” compared with these previous studies. In Ince et al's study, patients with submassive pulmonary embolism were included, and arterial blood samples were obtained while they were breathing room air to avoid interference from supplemental oxygen administration. This indicates that the population we studied was significantly different from that of Ince O. Our study population consisted of sepsis patients most of whom were on ventilator therapy, which can explain why D(A‐a)O_2_ was significantly higher in this population than in previous studies. In Pipitone et al's study, D(A‐a)O_2_ was obtained on admission to hospital. However, the study population was COVID‐19 patients, and the outcome was whether or not they had severe pneumonia. Therefore, the patients had D(A‐a)O_2_ measured on admission, and their lung function did not deteriorate at that time. This suggests that the specimens were likely collected and analyzed for blood gas in a situation where mechanical ventilation was not being used.

In our retrospective analysis, we first observed the relationship between D(A‐a)O_2_ and 28‐day death of patient with sepsis by using univariate and multivariate analyses, in which D(A‐a)O_2_ was used as an exposure variable and 28‐day death as an outcome variable with other variables being adjusted. We found that 28‐day mortality climbed with the increase of D(A‐a)O_2_ whether D(A‐a)O_2_ was used as a continuous or a categorical variable. We further conducted smoothed curve fitting by adjusting all other covariables and found out a nonlinear association between D(A‐a)O_2_ and the risk of 28‐day death in sepsis patient, in which a 5% increased risk of death was paired with a 10 mmHg rise of D(A‐a)O_2_ when D(A‐a)O_2_ was more than 300 mmHg. As we employed multiple statistical analyses and adjusted all other cofounders, we think our results are reliable.

Sepsis begins with an infection and progresses to multiple organ dysfunction via cytokine storm, among which respiratory system is vulnerably involved, mostly represented as a sepsis‐induced ARDS. Clinical data demonstrated that once sepsis patients was complicated with ARDS, their condition would inevitably deteriorate and their survival probability would also significantly be affected.^14^, ^15^ The important involved reasons, we guess, include the dysfunction of pulmonary diffusion capacity and mismatch of V/Q ratio, which are important pathophysiologic characteristics of ARDS. We did not know whether there were some sepsis‐induced patients included in our analysis, but it could be inferred from the distribution of data that D(A‐a)O_2_ values of many patients were more than 300 mmHg, a value obviously exceeding the normal threshold, implying many sepsis‐induced ARDS patients in our analysis. Therefore, we have the reason to think that patients with D(A‐a)O_2_ of more than 300 mmHg are actually these patients with diffusion dysfunction and/or V/Q mismatch, that is, sepsis‐induced ARDS. So the nonlinear relationship found in our result means that the more serious the sepsis‐induced ARDS, the higher the possibility of 28‐day death. Anyway, our reliable findings suggested that D(A‐a)O_2_ values are useful indicators effectively predicting the risk of 28‐day death in patients with sepsis.

Our work has the following advantages: (1) It has a large sample size and statistically significant power; (2) It employs a generalized summation model and a two‐piecewise linear model, both of which are advanced algorithms used to better determine the genuine association between D(A‐a)O_2_ and death.(3) The more covariate data, together with the presentation of multiple adjustment strategies and sensitivity analyses, ensure the robustness of the results and decrease the probability of chancing conclusions.

Meanwhile, there are some limitations in our analysis: (1) Given that this is a clinical retrospective study, it is inevitably subject to confounding factors. Nevertheless, we have systematically accounted for the confounding factors, and the robustness of the results has been examined by sensitivity analysis. (2) Due to the nature of observational studies, we can only observe relationships rather than determine cause and effect. (3) We can only account for detectable confounding, not unmeasurable puzzling, so extensive clinical research with stronger levels of evidence in larger populations is required to corroborate our findings. (4) As the population investigated in this study involves septic patients in the United States, researchers will have to exercise caution when extrapolating our findings to other populations. (5) Given that this study is a secondary data analysis based on a large, multicenter critical care database, we were unable to include patients based on definitions and clear criteria for sepsis and septic shock as in the actual clinical scenario. This could potentially result in some patients who meet the definition of sepsis or diagnostic criteria not being included. However, because it is not possible to determine whether this misclassification is related to the exposure variable as well as the outcome variable, the impact of this misclassification on our findings is unknown.

## 5 CONCLUSION

Our results demonstrated that D(A‐a)O_2_ has a nonlinear impacts on the risk of 28‐day death in patients with sepsis, meaning, the D(A‐a)O_2_ is a reliable indicator to predict the prognosis in this population.

## AUTHOR CONTRIBUTIONS

Ying Wang contributed to study concept and design and drafting of the manuscript. Lu Chen gained access to the database and is responsible of the data extraction. Yan He interpreted the data. Ying Liu, Jia Yuan, Hongying Bi, Qimin Chen, and Xianjun Chen helped with the data arrangement. Feng Shen contributed to the study concept, supervision and organized the final manuscript. All authors have read and approved the manuscript for publication.

## CONFLICT OF INTEREST STATEMENT

The author(s) declared no potential conflicts of interest with respect to the research, authorship, and/or publication of this article.

## ETHICS STATEMENT

The study has been approved by the Ethics Committee of Affiliated Hospital of Guizhou Medical University in accordance with the Helsinki Declaration.

## CONSENT FOR PUBLICATION

All authors consent to publicize the manuscript in the Internal Medicine Journal.

## NOMENCLATURE

D(A‐a) O_2_
: alveolar–arterial oxygen gradient
ARDS: acute respiratory distress syndrome
SOFA: Sequential Organ Failure Assessment

## Supporting information


**Table S1:** Adjustment vs no adjustment for Dopamine, Dexamethasone, Methylprednisolone, and Immunoglobulins use in logistic regression
**Table S2:** Adjustment vs no adjustment for Norepinephrine use in logistic regression
**Table S3:** Adjustment vs no adjustment for vancomycin, carbapenems, and cephalosporin use in logistic regression
