# Supplementary material for: Alveolar–arterial oxygen gradient nonlinearly impacts the 28‐day mortality of patients with sepsis: Secondary data mining based on the MIMIC‐IV database
Source: Clin Respir J. 2023 Apr 19;17(5):447–55. doi: 10.1111/crj.13614 (PMC10214571; doi:10.1111/crj.13614)
Supplement: Supplementary file 1 — Table S1: Adjustment vs no adjustment for Dopamine, Dexamethasone, Methylprednisolone, and Immunoglobulins use in logistic regression Table S2: Adjustment vs no adjustment for Norepinephrine use in logistic regression Table S3: Adjustment vs no adjustment for vancomycin, carbapenems, and cephalosporin use in logistic regression [file CRJ-17-447-s001.docx]

Supplemental table1：Adjustment vs no adjustment for Dopamine, Dexamethasone, Methylprednisolone, and Immunoglobulins use in logistic regression

| Exposure | Model 0  OR, 95%CI, p value | Model 1  OR, 95%CI, p value |
| --- | --- | --- |
| A-aDO2 per 10 change | 1.03 (1.023, 1.033) <0.001 | 1.03 (1.024, 1.033) <0.00001 |
| A-aDO2 (quartile) |  |  |
| Q1 | 1.0 | 1.0 |
| Q2 | 0.80 (0.69, 0.93) 0.003 | 0.81 (0.70, 0.94) 0.004 |
| Q3 | 0.94 (0.80, 1.10) 0.438 | 0.95 (0.81, 1.12) 0.534 |
| Q4 | 1.69 (1.41, 2.02) <0.001 | 1.71 (1.43, 2.05) <0.001 |
| P for trend | <0.001 | <0.001 |

Model 0: adjusted for variables that are presented in Table 1.

Model 1: Remove Dopamine, Dexamethasone, Methylprednisolone, and Immunoglobulins use from Model 0

Supplemental table2：Adjustment vs no adjustment for Norepinephrine use in logistic regression

| Exposure | Model 0  OR, 95%CI, p value | Model 1  OR, 95%CI, p value |
| --- | --- | --- |
| A-aDO2 per 10 change | 1.02 (1.02, 1.024) <0.001 | 1.03 (1.023, 1.033) <0.001 |
| A-aDO2 (quartile) |  |  |
| Q1 | 1.0 | 1.0 |
| Q2 | 1.02 (0.91, 1.15)>0.05 | 0.80 (0.69, 0.93) 0.003 |
| Q3 | 1.10 (0.98, 1.25)>0.05 | 0.94 (0.80, 1.10) 0.438 |
| Q4 | 1.65 (1.47, 1.85) <0.001 | 1.69 (1.41, 2.02) <0.001 |
| P for trend | <0.001 | <0.001 |

Model 0: adjusted for variables that are presented in Table 1.

Model 1: Model 3 + Norepinephrine use

Supplemental table3：Adjustment vs no adjustment for vancomycin, carbapenems, and cephalosporin use in logistic regression

| Exposure | Model 0  OR, 95%CI, p value | Model 1  OR, 95%CI, p value |
| --- | --- | --- |
| A-aDO2 per 10 change | 1.03 (1.023, 1.033) <0.001 | 1.03 (1.024, 1.033) <0.001 |
| A-aDO2 (quartile) |  |  |
| Q1 | 1.0 | 1.0 |
| Q2 | 0.80 (0.69, 0.93) 0.003 | 0.79 (0.69, 0.92) 0.002 |
| Q3 | 0.94 (0.80, 1.10) 0.438 | 0.93 (0.80, 1.10) 0.400 |
| Q4 | 1.69 (1.41, 2.02) <0.001 | 1.70 (1.42, 2.03) <0.001 |
| P for trend | <0.001 | <0.001 |

Model 0: adjusted for variables that are presented in Table 1.

Model 1: Removevancomycin, carbapenems and cephalosporin use from Model 0
